# Desiccation- and Saline-Tolerant Bacteria and Archaea in Kalahari Pan Sediments

**DOI:** 10.3389/fmicb.2018.02082

**Published:** 2018-09-20

**Authors:** Steffi Genderjahn, Mashal Alawi, Kai Mangelsdorf, Fabian Horn, Dirk Wagner

**Affiliations:** ^1^GFZ German Research Centre for Geosciences, Helmholtz Centre Potsdam, Section 5.3 Geomicrobiology, Potsdam, Germany; ^2^GFZ German Research Centre for Geosciences, Helmholtz Centre Potsdam, Section 3.2 Organic Geochemistry, Potsdam, Germany; ^3^Institute of Earth and Environmental Science, University of Potsdam, Potsdam, Germany

**Keywords:** saline pan, Kalahari, *Halobacteria*, *Gemmatimonadetes*, *Firmicutes*

## Abstract

More than 41% of the Earth’s land area is covered by permanent or seasonally arid dryland ecosystems. Global development and human activity have led to an increase in aridity, resulting in ecosystem degradation and desertification around the world. The objective of the present work was to investigate and compare the microbial community structure and geochemical characteristics of two geographically distinct saline pan sediments in the Kalahari Desert of southern Africa. Our data suggest that these microbial communities have been shaped by geochemical drivers, including water content, salinity, and the supply of organic matter. Using Illumina 16S rRNA gene sequencing, this study provides new insights into the diversity of bacteria and archaea in semi-arid, saline, and low-carbon environments. Many of the observed taxa are halophilic and adapted to water-limiting conditions. The analysis reveals a high relative abundance of halophilic archaea (primarily *Halobacteria*), and the bacterial diversity is marked by an abundance of *Gemmatimonadetes* and spore-forming *Firmicutes*. In the deeper, anoxic layers, candidate division MSBL1, and acetogenic bacteria (*Acetothermia*) are abundant. Together, the taxonomic information and geochemical data suggest that acetogenesis could be a prevalent form of metabolism in the deep layers of a saline pan.

## Introduction

Extreme environments were once thought to be incapable of sustaining a variety of life; however, organisms have developed various methods of adapting to the harshest of environments, ranging from hot springs and hydrothermal vents (Kallmeyer, 2017) to permafrost (Mitzscherling et al., 2017) and hypersaline lakes (Ventosa et al., 2011). The present study focuses on the microbial community structure and geochemical characteristics of two different saline pan sediments in the Kalahari Desert in southern Africa. Saline pans are common geomorphic formations in arid and sandy interdune desert systems. These pan structures have been described in many arid and semiarid environments, such as Australia (Magee et al., 1995), China (Bowler et al., 1986), Africa (Lancaster, 1986; Goudie and Wells, 1995), and the southwestern United States (Handford, 1982), but they also exist in cold drylands, such as in Antarctica (Lyons et al., 1998). The drylands comprise the major terrestrial biome of the Earth’s land surface (Reynolds et al., 2007). Due to the desiccation of surface waters and high evaporation rates, aridification in the Kalahari region is a fast process occurring on a seasonal scale. High aridity and daily temperature fluctuations together with intense solar radiation contribute to an extreme habitat for the living organisms. The key microorganisms involved in the biogeochemical cycles of saline sediments and a large number of microbial processes, such as nitrogen fixation and sulfur metabolism, have been insufficiently characterized (Keshri et al., 2013).

All over the world, hypersaline environments – including athalassohaline lakes, evaporation ponds, deserts, and hypersaline environments with marine origins – are inhabited by a variety of microorganisms (DasSarma and Arora, 2001). Halophiles are salt-loving organisms from all three domains of the biological classification of life. They are adapted to grow in high-salt ecosystems, although their overall microbial diversity decreases with increasing salt concentrations (Oren, 1999). Prokaryotes are adapted to resist osmotic stress caused by the high concentration of ions in the external environment by using two main adaptive strategies. To prevent desiccation, first, the “high-salt-in” strategy enables the microorganisms to accumulate potassium and chloride in their cytoplasm. Second, the “low-salt, organic-solutes-in” strategy refers to the accumulation of organic osmotic solutes (Ma et al., 2010), such as ectoine, betaine, and sugars. In saline environments, the microbial life forms face thermodynamic limitations and hence require an appropriate mechanism of osmotic adaption and a form of metabolism that yields enough energy for biomass synthesis and osmoregulation (Oren, 1999, 2011). The majority of halophiles found in salt deposits are archaea, while bacteria form the minority groups (McGenity et al., 2000). The archaea that require the most saline conditions fall under the class *Halobacteria* (Oren, 2008; Youssef et al., 2012). These *Halobacteria* are often found in a diverse range of hypersaline environments, such as salt marshes (Bowen et al., 2012), solar salterns (Sørensen et al., 2005), and hypersaline soils (Crits-Christoph et al., 2013; Keshri et al., 2013). Halophiles within the bacterial community are known to exist in the phyla *Cyanobacteria*, *Proteobacteria*, *Bacteroidetes*, *Firmicutes, Chloroflexi*, and *Actinobacteria* (Oren, 2008).

Several bacterial species have been isolated from the hypersaline Lake Chaka in China via culture-dependent techniques (Jiang et al., 2006), whereas other studies have used molecular methods, such as 16S rRNA gene clone library sequencing, denaturing gradient gel electrophoresis (DGGE), or terminal restriction fragment length polymorphism (T-RFLP) band sequencing, and have highlighted the idea that the microbial diversity significantly varies between different saline pan systems (Lefebvre et al., 2006; Montoya et al., 2013). In recent years, the next-generation sequencing technologies have expanded our knowledge of the diversity and composition of microbial communities in saline ecosystems (Bowen et al., 2012; Youssef et al., 2012). In the present study, we applied Illumina 16S rRNA gene sequencing and geochemical methods to analyze the microbial diversity and the distribution patterns in Witpan in northwestern South Africa. In a second step, we compared the microbial communities and geochemical features of Witpan with those of the Omongwa pan (Genderjahn et al., 2018). This comparison allowed the identification of the microbial key community occurring at both sites as well as site-specific specialists that occur in southern African saline pans. Multivariate statistics were applied to identify the major community-shaping environmental factors. This study aims to provide a better understanding of the saline and dry habitats and their diverse microbial ecosystems.

## Materials and Methods

### Study Site and Sampling

In the Kalahari region, precipitation is regulated by the seasonal shift of the Intertropical Convergence Zone and the migration of the westerlies in the Southern Hemisphere (Ahrens and Samson, 2010). Seasonally strong precipitation leads to transient runoff into ephemeral rivers, and pans are temporally filled with water. Pans (also called playa) are the predominant geomorphic feature of the Kalahari. They are closed systems in terms of their surface hydrology because they have no surface outflow. The high evaporation exceeds the rainfall during all months (Lancaster, 1976; Goudie and Wells, 1995); therefore, these bodies of water are often highly saline (Shaw and Bryant, 2011). The hydrological input comes from direct precipitation, surface, or subsurface inflow, and standing surface water occurs ephemerally.

**FIGURE 1:**
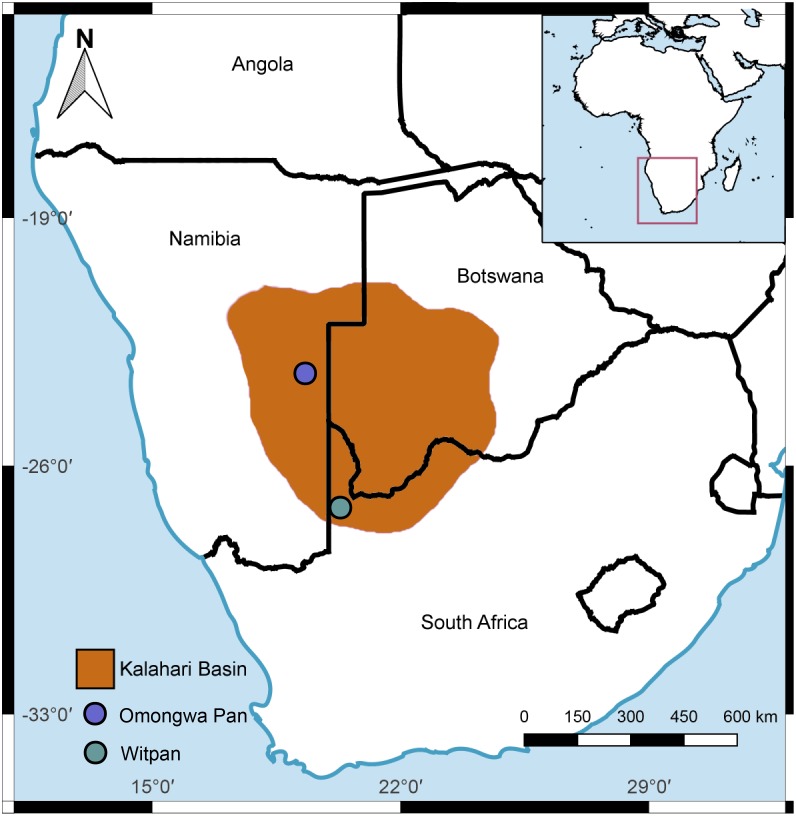
Map of the study sites: Witpan (S 26° 40, 658′ E 020° 09, 45′) in South Africa, Omongwa pan (S 23° 42, 59′ E 019° 22, 15′) in eastern Namibia, Kalahari Desert. Quantum GIS 2.18 was used to produce the map (http://www.qgis.org).

In autumn 2013, a field campaign to southern Africa was conducted in cooperation with the Helmholtz Centre Potsdam GFZ German Research Centre for Geosciences, the German Centre for Marine Biodiversity Research (DZMB) – Senckenberg am Meer, and the Institute for Chemistry and Biology of the Marine Environment (ICBM) of the University Oldenburg, Germany. Sample material was collected from the pan sediments of Witpan (S 26° 40, 658′ E 020° 09, 45′) in northwestern South Africa and from the Omongwa pan (S 23° 42, 59′ E 019° 22, 15′) in Namibia in the western Kalahari (**Figure 1**). The Witpan deposits display great variability in grain-size distribution. Silt and evaporite crystals dominate the top layers (0–14 cm), followed by a mixture of silt and sand. Fine- and medium-grained sand make up the largest proportion of the sediment, which is between 25 and 119 cm. Clay and silt occur between 119 and 180 cm and dominate Witpan sediment composition at this depth interval (see **Supplementary Figure S1**). The groundwater table lies at a depth of 230 cm. The Omongwa pan is characterized by the presence of little organic matter, dispersed evaporated crystals, and low-porosity, fine-grained sediments, which mostly consist of silt and gypsum crystals (**Supplementary Figure S1**, Schüller et al., 2018). During sampling, the Omongwa pan was covered by a thin saline crust. According to Milewski et al. (2017), the main mineralogy components of the top layer are halite (NaCl, 94%) and gypsum (CaSO_4_⋅2H_2_O, 3%).

Samples were taken from a 50 cm deep trench excavated from the sediment. Between the surface and the top 15 cm, samples were taken at 3-cm intervals, followed by 5-cm intervals between 15 and 50 cm. A short core (50–180 cm) was drilled with an Eijkelkamp hand auger and sampling continued at 15-cm intervals in this section. The samples for molecular biological analyses were taken from the inner parts of the drill core using a sterilized spatula. The samples for biomarker analyses were immediately frozen in liquid nitrogen, and the samples for molecular studies were cooled during the field work and were kept at -24°C after their arrival at GFZ Potsdam.

### Quantification of Phospholipid-Derived Fatty Acids

The samples were freeze-dried and homogenized. They were ground using a disk-mill with stainless steel grinding set and extracted with a modified Bligh and Dyer (1959) method that included solvent extraction and phase separation. A sample preparation method after Zink and Mangelsdorf (2004) was used to separatethe total the lipid extract into a low polar fraction, a free fatty acid fraction, a glycolipid fraction, and an intact phospholipid (PL) fraction. The internal standard 1-myristoy-(D27)-2-hydroxy-sn-glycerol-3-phosphocholine was added to quantify the phospholipids. Half of the PL fraction was saponicated to obtain the phospholipid-derived fatty acids (PLFAs) (Müller et al., 1990). The samples were measured on a DSQ Thermo Scientific Quadrupole mass spectrometry (Thermo Fisher Scientific) coupled with a gas chromatograph (TRACE GC Ultra, Thermo Fisher Scientific) equipped with a cold injection system BPX5 (SGE) column (50 m length, 0.22 mm inner diameter, 0.25 μm film thickness). The gas chromatograph (GC) was run in splitless mode with the following settings: injector temperature from 50 to 300°C with 10°C/s, oven temperature from 50°C (1 min isothermal) with a heating step with a rate of 3°C min^-1^ to 310°C finally held for 30 min. Helium was utilized as a carrier gas at a constant flow rate of 1 mL min^-1^. The gas chromatography-mass spectrometer (GC-MS) was run in electron impact (EI) ionization mode at 70 eV. Full-scan mass spectra were recorded from m/z 50–650 amu at a scan rate of 1.5 scans s^-1^.

### Sediment Properties

Since the sediment samples for measuring the ionic composition contained too little pore water, they were leached, as indicated by Blume et al. (2011). An aliquot of 5 g of each sample was suspended in 25 mL of deionized water, shaken for 90 min, and centrifuged to remove all the solids. The ion concentrations of anions and organic acids were measured by ion chromatography (IC). The leached samples were investigated for the following ions: chloride, nitrate, sulfate, acetate, and formate. The specifics of the method to detect organic acids have been described by Vieth et al. (2008); for inorganic acids, see Noah et al. (2014). Analytical settings are shown in the **Supplementary Table S1**. Furthermore, the total organic carbon (TOC) content of each sediment sample was measured according to DIN EN 1484-H3 by Potsdamer Wasser und Umweltlabor GmbH & Co. KG, Germany.

### DNA Extraction and Preparation of Next-Generation Sequencing

The total genomic DNA was extracted in triplicate from 0.3–0.5 g sediment material using the Power Soil^TM^ DNA Isolation Kit (Mo Bio Laboratories Inc., Carlsbad, CA, United States) in compliance with the company’s protocol. To enhance the efficiency of the DNA extraction, all the samples were heated up for 10 min to 70°C after step four. The DNA triplicates were pooled for downstream analysis.

The hypervariable region V4 of the 16S rRNA gene was targeted for a subsequent amplification using the primer pair 515F and 806R (Caporaso et al., 2011). The polymerase chain reactions (PCR) were carried out at least in technical triplicates. The 50 μl PCR reaction mix consisted of 25 μl Mango-Mix (including a MangoTaq^TM^ DNA Polymerase, MgCl_2_, and ultra-pure dNTPs manufactured by Bioline GmbH, Luckenwalde, Germany) and 1 μl of each primer (10 mM), and 5 μl of template. The mix was filled up to 50 μl with PCR-clean water (MO BIO Laboratories, Inc., Carlsbad, CA, United States). Afterward, the PCR products were run on a 1% agarose gel in a 1× Tris-acetate-EDTA buffer stained with GelRed^TM^ Nucleic Acid Gel Stain (Biotium, United States) to check the samples for sequencing. The PCR products were pooled and purified by the Genomic DNA Clean & Concentrator^TM^-10 (Zymo Research, United States) and quantified by the Qubit Fluorometer (Invitrogen^TM^, Thermo Fisher Scientific, United States) for library preparation. The samples were sent to the Illumina MiSeq platform Eurofins Genomics, 85560 Ebersberg, Germany. Sequence libraries were generated by a 2 × 250-bp paired-end approach. The primer sequence consisted of a 6-bp tag and spanned the V4 region. Data were received as raw FASTQ files and deposited into the European Nucleotide Archive (sample accession: ERS1599441 – ERS1599469).

### Processing Next-Generation Sequencing Data

Assembling the reads was performed using PEAR (Zhang et al., 2014). Standardizing the nucleotide sequence orientation, trimming (see also **Supplementary Table S2**) and filtering the low-quality sequences were performed using Trimmomatic, which has been described in detail by Bolger et al. (2014). Afterward, all the chimeras were removed, and the sequences were clustered into OTUs (QIIME pipeline). A taxonomic classification was assigned by the SILVA database (Version 128) (Quast et al., 2013) with a cutoff value of 97% using the QIIME open-source software package and by choosing the open-reference OTUs (Caporaso et al., 2010). The following filters were applied: removing singletons and eliminating all operational taxonomic units (OTUs), which had an occurrence of less than 0.5% in each sample. Overall, the diversity was estimated using the statistic program PAST 3.15 with a taxonomic method (Shannon index, H). The Shannon index is a phylotype-based method created with OTU grouping (McCann et al., 2016). Statistics were carried out using CANOCO 5. The species response to environmental variations was modeled with the help of a canonical correspondence analysis (CCA) (Ramette, 2007).

### Quantitative Polymerase Chain Reaction Analysis of Archaeal and Bacterial SSU rRNA Genes

A quantitative polymerase chain reaction (qPCR) was used to quantify the total bacterial and archaeal abundances (**Supplementary Figure S2**). For bacteria, the forward primer Eub 331-F 5′-TCCTACGGGAGGCAGCAGT-3′ and the reverse primer Eub 797-R 5′-GGACTA CCAGGG-TATCTAATCCTGTT-3′ (Nadkarni et al., 2002) were used to amplify the fragments from the bacterial SSU rRNA genes. The quantification of the archaeal 16S rRNA gene was based on the primers A751F 5′-CCgACGGTGAGRGRYGAA-3′ and UA1204R 5′-TTMGGGGCATRCIKACCT-3′ (Baker et al., 2003). All the qPCR analyses were performed in analytical triplicates in a thermal cycler (CFX Connect^TM^ Real-Time PCR Detection System, Bio-Rad Laboratories, United States) instrument using the polymerase iTaq^TM^ Universal SYBR^®^ Green Supermix (Qiagen). Each PCR essay contained 12.5 ml of Universal SYBR^®^ Green Supermix SYBR^®^ – including the polymerase iTaq^TM^, 0.5 μl of each primer (20 mM), and 5 μl template (diluted, 1:7) – and was filled up to 25 ml with PCR-clean water (MO BIO Laboratories, Inc., Carlsbad, CA, United States). After the initial denaturation phase of 30 s at 95°C, the annealing phase followed at 58.5°C for 1 min. Elongation was performed at 72°C for 30 s and then at 80°C for 3 s. In total, 40 cycles were run. To generate a standard curve, known dilutions (10^1^–10^7^ gene copies) of the target fragments amplified from *Methanosarcina barkeri* (for archaea) and *Bacillus subtilis* (for bacteria) were used. Finally, the melting curve analyses were performed to ensure correct amplification.

## Results

### Abiotic and Biotic Parameters of Witpan and Omongwa Pan

**FIGURE 2:**
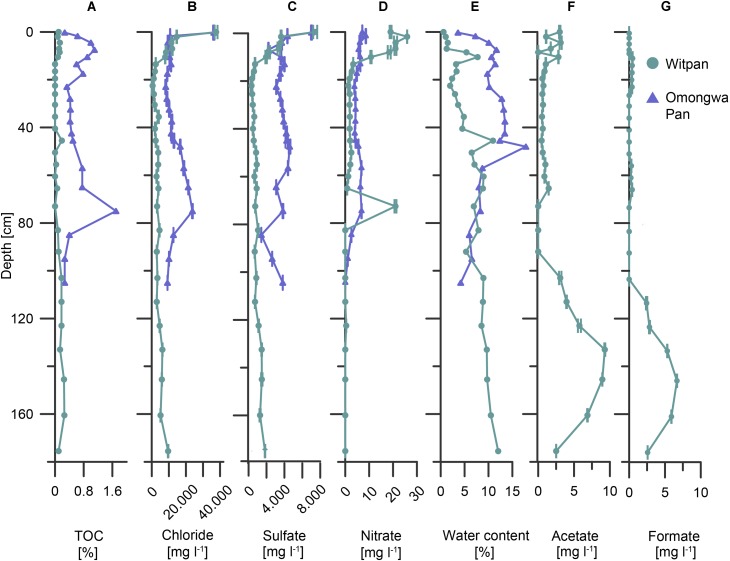
Abiotic and biotic parameters of Witpan (turquoise) and the Omongwa pan (purple) with depth. **(A)** Total organic carbon (TOC), **(B)** chloride, **(C)** sulfate, **(D)** nitrate, **(E)** water content, **(F)** acetate, and **(G)** formate [**(B–D,F,G)** were obtained from sample leaching]. Note different x-scales.

In Witpan, the values of the deposits of the TOC are rather low and range from 0.03 to 0.26 wt% (**Figure 2A**). Between 14 and 50 cm, the TOC content is below the detection limit (0.01 wt%). A slight increase in TOC was detected from 80–160 cm. In contrast, the TOC of the Omongwa pan ranges from 0.3 to 1.7 wt%, with the highest values in the top 20 cm and a maximum value at a depth of 75 cm (**Figure 2A**).

Chloride, sulfate, and nitrate are the predominant anions in the near-surface layers of both pans. In Witpan sediments, the concentrations increase up to 38,000 mg l^-1^ of chloride, up to 7,500 mg l^-1^ of sulfate, and up to 26 mg l^-1^ of nitrate (**Figures 2B–D**). Below the surface layer, chloride and sulfate concentrations decrease to 480 and 370 mg l^-1^, respectively, before beginning to slightly but progressively increase again to 9,560 mg ^-1^ (chloride) and 1,880 mg l^-1^ (sulfate) at 175 cm. The nitrate concentration is quite low (around 2 mg l^-1^) or absent between 120 and 175 cm, with an exception at a depth of 70 cm (21 mg l^-1^). In Omongwa pan sediments, the chloride concentrations range from 8,200 to 36,300 mg l^-1^, and the sulfate concentrations vary between 1,500 and 7,100 mg l^-1^. Both concentrations are greatest in samples close to the surface, followed by an abrupt decrease with depth. Between 45 and 80 cm, chloride increases again. The nitrate concentrations in the Omongwa pan decrease with depth and are significantly lower (up to 8.6 mg l^-1^, **Figure 2D**) than those of chloride or sulfate. The water content of Witpan is around 0.6% in the top layers and increases slowly but more-or-less steadily with depth up to ∼12%. In the Omongwa pan sediments, the water content is around 4% in the upper layer and increases to 18% at a depth of 50 cm. After this peak, the water content decreases again with depth to 4% (**Figure 2E**). Low molecular weight acids such as acetate and formate could only be detected in Witpan in varying concentrations between 0.2 and ∼9 mg l^-1^ (**Figures 2F,G**). The acetate and formate concentrations increase at a depth between 100 and 160 cm compared with the upper part of Witpan.

### Microbial Community Composition of Witpan

**FIGURE 3:**
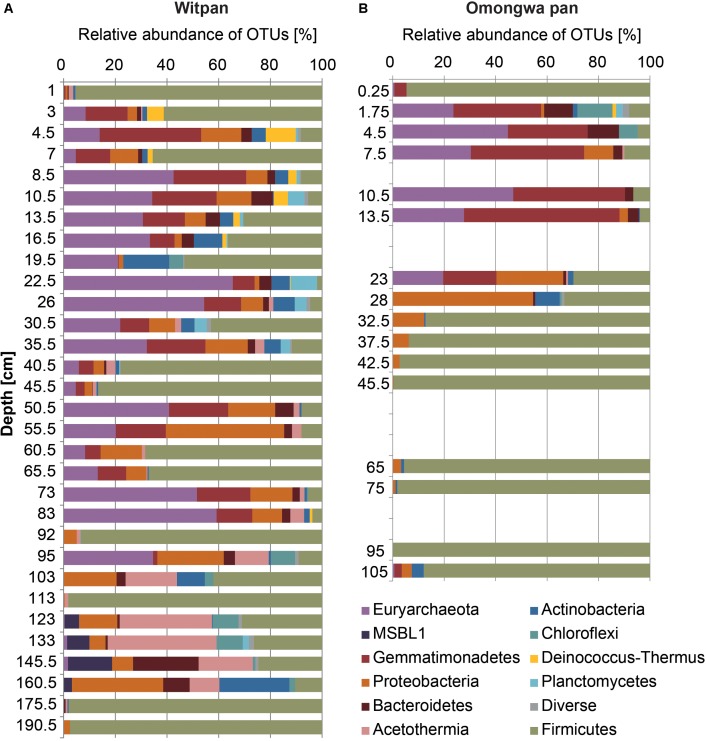
Microbial communities of Witpan **(A)** and Omongwa pan **(B)**. Depth profile shows different bacterial and archaeal phyla obtained using high-throughput Illumina sequencing. Color codes are indicated on the right.

In the dataset described here, 407 different OTUs were found to be distributed between the Omongwa pan and Witpan. Detailed taxonomic information was assigned to the relative abundance (rel. ab.) of OTUs within the microbial community. Classified sequences from Witpan showed forms of life from 20 phyla, with bacteria accounting to 36–100% of the microbial community (**Figure 3A**). The majority of archaeal sequences could be assigned to *Euryarchaeota* (17 OTUs) and detected almost throughout the entire depth profile. Fourteen OTUs of *Euryarchaeota* belong to the class *Halobacteria*, including the genera *Halobiforma*, *Natronococcus*, *Halococcus*, *Halomicrobium*, and a high proportion of uncultured genera. *Halobacteria* were observed from the near-surface layers down to 103 cm and represent up to 65% (at 22.5 cm) of the microbial community. Archaeal sequences in deeper layers (from 123 to 160 cm) are affiliated with candidate division MSBL1 (2 OTUs) and form up to 10% of the combined bacterial and archaeal community. Only at 133 cm was one archaeal OTU of *Hadesarchaea* detectable (less than 1.8% of the community).

Sequences of *Firmicutes* were detected throughout the entire depth profile. Several layers are dominated by *Firmicutes* such as the surface layer and layers at 40 to 45.5 cm, 92 cm, 113 cm, and 175 cm, where they represent more than 95% of the community. The genus *Bacillus* dominates the phylum *Firmicutes* and occurs in every depth range. In addition, *Clostridia* could be found mainly between 103 and 160.5 cm (up to 13% rel. ab.). The bacterial sequences of *Gemmatimonadetes* (7 OTUs) are abundant from the top layers down to 95 cm and form up to 38% of the microbial community (e.g., at 4.5 cm). Four orders could be identified: *Longimicrobia* (2–16.5 cm), S0134 (13.5–16.5 cm), PAUC43f (19.5–83 cm), and BDS-11 (22–83 cm). *Bacteroidetes* can be assigned to 6 different classes including *Bacteroidia* (1 OTU), *Cytophagia* (5 OTUs), *Flavobacteria* (3 OTUs), *Sphingobacteria* (2 OTUs), and *Bacteroidetes Incertae Sedis* Order III (5 OTUs, formerly assigned to *Rhodothermales*). Down to 83 cm, *Bacteroidetes Incertae Sedis* (1–8% rel. ab. of all sequences) dominates the phylum *Bacteroidetes*, whereas in the deep layers from 123–160 cm, *Bacteroidia* are predominant (up to 23% of the microbial community). *Proteobacteria* (59 OTUs) are present all over the depth profile and consists of *Alpha*-, *Beta*-, *Gamma*-, and *Delta-proteobacteria*. At a depth of 123–175 cm, the families *Methylophilaceae*, *Comamonadaceae*, *Desulfohalobiaceae*, and the order *Xanthomonadales* become predominant within the phylum *Proteobacteria*. In layers from 24–34 cm, the order *Rhodospirillales* comprises 1–4% of the microbial community. The order *Rhizobiales* (including mostly *Salinarimonas* and *Methylobacteriaceae*) occurs infrequently, and sequences could mainly be detected between 2 and 7 cm, forming 1 to 4% of the community. In the deep layers from 95–175 cm, *Rhizobiales* make up to 1% of the microbial community. *Actinobacteria* sequences that can be assigned the class *Nitriliruptoria* (6 OTUs) were found between 7 and 45.5 cm in Witpan (1–4% rel. ab.). Among the sequences assigned to *Actinobacteria*, *Acidimicrobiales* (3 OTUs) occur infrequently between 2 and 83 cm (0.5–5%), and *Actinomycetales* (13 OTUs) could be detected at 13.5–19.5 cm, 40.5 cm, 95 cm, and 160 cm, with relative abundances between 0.4–8%.

Sequences affiliated with the phylum *Chloroflexi* and the phylum *Acetothermia* – formerly known as OP1 (Rinke et al., 2013; Nigro et al., 2016) – become dominant in the deep layers, especially from 95–160 cm. *Chloroflexi* belong to the class *Dehalococcoidetes* (5 OTUs) and comprise the candidate groups GIF 9 and MSBL5. *Dehalococcoides* sequences occur especially often in the deeper sections (92–145 cm, 2–8% of the total sequence abundance). Sequences related to *Acetothermia* are highly abundant between 95 and 160 cm and make up 12–40% of the total microbial community.

The phylum *Deinococcus-Thermus* (1 OTU) occurs in the upper layers between 2 and 16.5 cm (2–12%), and the OTUs belong to the genus *Truepera*. *Planctomycetes* (4 OTUs) are present from 4.5 to 40 cm, with up to 10% of the microbial community at a depth of 23 cm. *Planctomycetes* are represented by the classes of *Phycisphaerae* (3 OTUs) and *Planctomycetia* (4 OTUs). Additionally, *Spirochaetaceae* sequences (1 OTU) could be found between 26 and 35 cm and make up less than 2% of the microbial community. The abundance of recovered *Verrucomicrobia* (2 OTUs) occurs between 4.5 and 10 cm as well as between 95 and 103 cm depth and form ∼1% of the microbial community. Fourteen OTUs could not be assigned.

### Microbial Community of Omongwa Pan

In total, 121 OTUs from 13 different bacterial and archaeal phyla were detected using high-throughput sequencing. Absolute read counts were transformed to relative abundances in order to standardize the data. *Firmicutes* dominate the depth profile of the Omongwa pan from 32.5–105 cm as well as the surface layer (85–98% of the microbial community, **Figure 3B**). Within this phylum, three different orders are represented: *Lactobacillales* (2 OTUs), *Thermoanaerobacterales* (1 OTU), and *Bacillales* (2 OTUs). *Bacillales* can be assigned to the genus *Bacillus*. Between 1.75 and 23 cm, a high proportion of *Euryarchaeota* (20–47%) and *Gemmatimonadetes* (21–61%, **Figure 3B**) were detected. Three orders of *Gemmatimonadetes* were identified: *Longimicrobia*, PAUC43f, and BDS-11. Around 25 OTUs of *Euryarchaeota* can be assigned to *Halobacteria* (Gupta et al., 2016); hence, a few were identified at the genus level: *Natronomonas* (3 OTUs), *Natronococcus* (1 OTU), *Haloterrigena* (1 OTU), and *Halorhabdus* (2 OTUs). The OTUs assigned to *Proteobacteria* dominate between 23 and 28 cm (26–54%, **Figure 3B**). A high abundance of *Bacteroidetes* (2–14%) sequences were detected between 2 and 30 cm (**Figure 3B**). Almost all *Bacteroidetes* can be attributed to the family *Rhodothermaceae* (2 OTUs), whereby three OTUs were identified as being from the family *Salinibacter*. Additionally, the phyla with less than 10% of relative abundance were found (**Figure 3B**), such as *Actinobacteria* (7 OTUs), *Chloroflexi* (3 OTU), *Fusobacteria* (1 OTU), *Planctomycetes* (1 OTU), *Deinococcus-Thermus* (1 OTU), *Acetothermia* (1 OTU), slight traces of *Thaumarcheaota* (1 OTU), and 2 OTUs of unassigned phyla.

### Statistical Analyses and Description of Core Taxa Among Two Different Continental Pans

**FIGURE 4:**
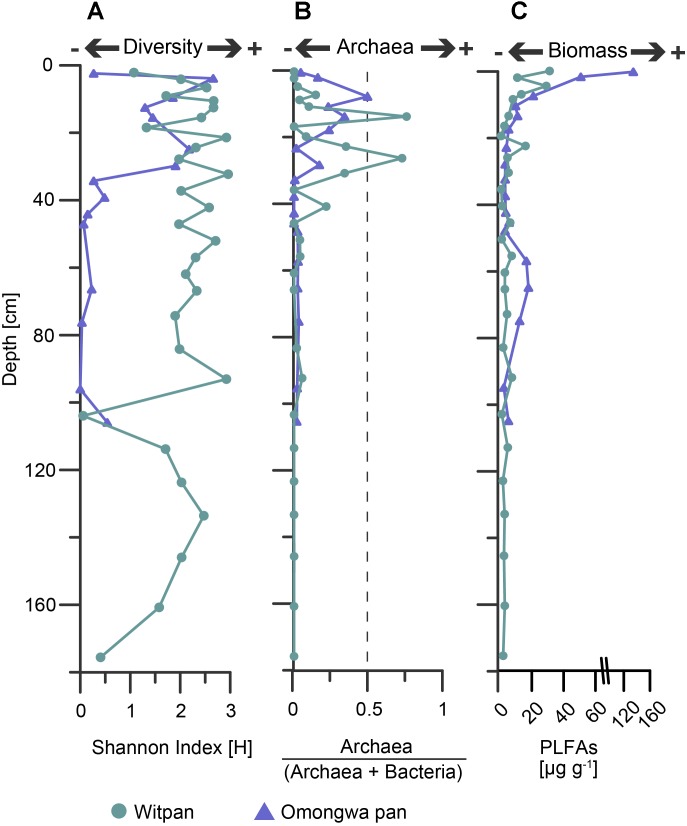
**(A)** Shannon index [H] based on OTU sequencing data, **(B)** abundance of archaea vs. archaea + bacteria based on archaeal and bacterial 16S rRNA genes 16S, and **(C)** biomass input was calculated from phospholipid fatty acids (PLFAs). Witpan, turquoise; Omongwa pan, purple.

The Shannon index (H-index) demonstrates the species diversity within a community (Colwell, 2009) and was calculated based on OTU sequences. The h-index of Witpan decreases with depth. At 103 and 175.5 cm, the h-index is below 0.4. For all other depths, the h-index varies between 1 and 3. In the Omongwa pan, the h-index varies from 0 to 2.2, and two distinct microbial communities could be observed. Within the top layers (1.8–28 cm), the community is characterized by higher species diversity (Shannon index Ø 1.8), whereas the second community (32.5–105 cm) consists of only a few species (Shannon index Ø 0.19, **Figure 4A**).

A ratio (archaea / [archaea + bacteria]) based on the total abundances of bacterial and archaeal 16S rRNA genes was calculated (**Figure 4B**). In both the pan sediment profiles, the abundance of archaea decreases with increasing depth. In the Omongwa pan, a larger number of archaea within the top 23 cm (up to 49%, **Figure 3B**) could be observed. The microbial communities of Witpan are partly dominated by bacteria that comprise 28–100% of the sediment. In the near-surface layers (0–7 cm) archaea are less abundant (0–14%). Archaea become more abundant and partly dominate the microbial community at 13.5 cm and 26 cm, where they comprise up to 75% of the sediment (**Figure 3A**). The archaeal SSU genes were not detected in the deeper layers of Witpan (92–175 cm, **Figure 4B**).

**FIGURE 5:**
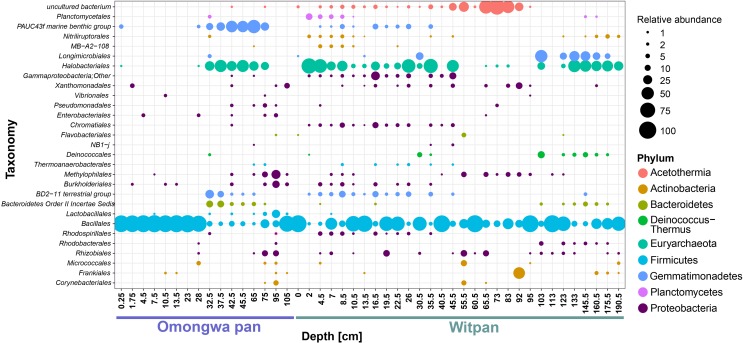
Identification of shared OTUs between Omongwa pan and Witpan is based on order level taxonomic distribution in sediment depth profile. Bubble size indicates the relative abundance. Different phyla are indicated via color-coding.

At the order level, 29 shared taxa from Witpan and the Omongwa pan (**Figure 5**) are distributed among nine different phyla. This core community is dominated by *Bacillus*, *Halobacteria*, and *Gemmatimonadetes* (**Figure 5**). The remaining sequences can be assigned to *Actinobacteria*, *Bacteroidetes*, and *Proteobacteria*. Within the core community, a few sequences closely related to *Planctomycetes* and *Acetothermia* could also be identified.

**FIGURE 6:**
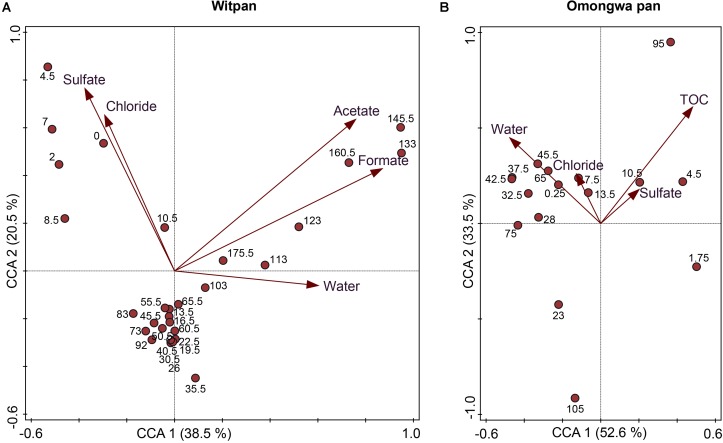
Canonical correspondence analysis (CCA) correlates the environmental parameters (chloride, sulfate, TOC, water content, acetate, and formate) with OTU sequencing data and different sampling depths (cm) in **(A)** Witpan and **(B)** Omongwa pan. All *p*-values are <0.05.

Statistical analysis was applied to identify the correlations between the environmental data and the identified OTUs using CCA (**Figure 6**). The CCA log file of Witpan revealed a total variation of 6.079, while explanatory variables account for 25.4% of the total variance in bacterial and archaeal distribution. Axes 1 and 2 explain 38.5 and 20.5% of the total variance, respectively. The CCA results of Witpan reveal a grouping of deep layers (103–175.5 cm) according to the acetate, formate, as well as water content. The upper layers (0–19.5 cm) of Witpan are characterized by high chloride and sulfate concentrations. The microbial variation of the Omongwa pan is also explained by the environmental parameters using a CCA plot. 31.4% of the total variance in bacterial and archaeal distribution can be accounted for by environmental parameters (total variation: 3.403). Axes 1 and 2 explain 52.6 and 33.5% of the total variance, respectively. Chloride and sulfate concentrations are related to the surface layer and to the deeper layers (42.5–105 cm) of the Omongwa pan. In contrast, the layers between 25 and 75 cm can be grouped according to the water content. *P*-values of all the descriptive variables are below 0.05.

### Analyses of Phospholipid Fatty Acids (PLFAs) From Witpan and Omongwa Pan

In the Witpan sediments, saturated (C_14:0_ to C_20:0_), branched (*iso/anteiso*-_C15:0_, *iso*-C_16:0_, *iso/ai*-C_17:0_, 10Me-10Me-C_16:0_), unsaturated (C_16:1ω9_, C_16:1ω7_, C_16:1ω5_, C_18:1ω7_, and C_18:1ω9_), and cyclopropyl (cy-C_17:1_ and cy-C_19:1_) PLFAs were identified (Genderjahn et al., 2018). Molar amounts (nmol g^-1^ of dry weight, dw) of the saturated PLFAs were used as an estimation of the microbial biomass. The highest values were found in the surface layer (31 μg g^-1^) and decreased with depth down to 0.9 μg g^-1^ at 35.5 cm and 2 μg g^-1^ at 175 cm (**Figure 4C**).

In the Omongwa sediments, saturated (C_12:0_ to C_22:0_), branched/saturated (*iso*-C_14:0_, *iso/ai*-C_15:0_, *iso*-C_16:0_, *iso/ai*-C_17:0_, 10Me-C_16:0_), unsaturated (C_16:1ω7_, C_16:1ω5_, C_18:1ω7_, and C_18:1ω9_, branched C_19:1_), and cy-C_17:1_ PLFAs were identified. The highest concentration of PLFAs was identified in the uppermost layers at a sediment depth from 0–3 cm (135 μg g^-1^) and decreased significantly with depth (down to 3.2 μg g^-1^ at 32.5 cm). A slight increase in the estimated biomass based on the saturated PLFA concentrations was observed between 60 and 80 cm (**Figure 4C**).

## Discussion

### Kalahari Pans as a Habitat for Microorganisms

Desert environments are ecosystems with limited nutrient availability and high desiccation potential (Pointing and Belnap, 2012). A detailed analysis of the Kalahari pan sediments provide insights into the microbial abundance and diversity. Both Witpan and the Omongwa pan (see also Genderjahn et al., 2018) are characterized by a low amount of total organic carbon (**Figure 2A**), but display differences in their texture (see **Supplementary Figure S1**). The Omongwa pan consists mainly of silt, whereas the Witpan sediments are more heterogonous geneous in terms of lithological texture. Witpan is primarily composed of a mixture of clay and silt in the near-surface layers followed by sandy layers from 25–120 cm, while the deeper layers are dominated by clay. Sand fractions in Witpan can act as a “skeleton,” and water can quickly infiltrate the deeper layers of the sediment. The coarser sediment structure of Witpan might explain the fluctuations in the microbial richness found there (**Figure 4A**). Sediment texture, in terms of the composition of clay, silt, and sand, as well as the pore size distribution, affects the kinetic of the available nutrients as well as electron acceptors and water retention (Rhodes, 2012). Our data on Witpan deposits suggest an increased substrate potential (electron donors and acceptors) for microorganisms in the near-surface layers and in deeper layers from 110–180 cm, whereas in the Omongwa pan, the formate and acetate were not measurable (**Figures 2F,G**). Buried organic matter is the main source of carbon and energy for microorganisms in the sedimentary systems (Schaechter, 2009), and acids with low molecular weight, such as formate and acetate, are the key intermediates in microbial heterotrophic metabolism (Schulz and Zabel, 2013). Based on our CCA analyses, the microbial community composition is influenced by salinity and the availability of water in Witpan and in the Omongwa pan (**Figure 6**). In arid systems, the salinity naturally increases due to evaporation after rainfall (Youssef et al., 2012). The dominance of chloride and sulfate in the investigated pans (**Figures 2B,C**) indicates that salinity is a key factor that affects the pan microbiomes, and specialized microorganisms were found to occur.

### Microbial Community Structure in Witpan Deposits

The Kalahari pans are extreme habitats in the sense that they are highly limited in moisture and salinity is usually extremely high. The present study provides insights into the complex microbial community structure of Witpan for the first time. *Firmicutes* is the most abundant phylum in the present dataset (up to 95%, **Figure 5**). This taxon is characterized by fast spore germination and a short doubling time. Among the sequences assigned to *Firmicutes*, the dominant genus is *Bacillus*. A large number of *Bacillus* have been isolated from saline and alkaline soil habitats (Ren and Zhou, 2005; Jiang et al., 2006). In the deep anaerobic layers of Witpan, *Firmicutes* of the families *Natranaerobiaceae* and *Syntrophomonadaceae* occur. *Natranaerobiaceae* are halophilic alkalithermophilic bacteria and are model organisms for evaluating theories on the origin of life (Mesbah et al., 2007), including the hypothesis that life evolved in the shallow, heated saline and alkaline pools (Wiegel and Michael, 2002). *Syntrophomonadaceae* can convert fatty acids produced by fermentative bacteria to acetate, formate, or hydrogen, which can then be consumed by methanogens (Dworkin et al., 2006). In general, the syntrophic bacteria are found when the organic matter is degraded and inorganic electron acceptors are missing.

*Gemmatimonadetes* are found in low frequency in all types of soils, including marine and lake sediments (DeBruyn et al., 2011; Fierer et al., 2012). They are predominant in hyper- and semi-arid soils with very low biomass, as in the Sahara Desert (Meola et al., 2015), the Atacama Desert (Schulze-Makuch et al., 2018), as well as Arctic polar deserts (McCann et al., 2016) and Antarctic glacier forefields (Bajerski and Wagner, 2013). In Witpan, *Gemmatimonadetes* form up to 38% of the microbial community and occur between 0–95 cm depth (**Figure 3A**). Slowly growing *Gemmatimonadetes* (Zhang et al., 2003) are important colonists that adapt well to low soil moisture (DeBruyn et al., 2011), pointing to a tolerance for desiccation.

Archaea comprise up to 65% of the entire microbial community. The majority of the archaeal sequences are related to halophilic archaea, namely *Halobacteria*. These findings are consistent with several other studies on the archaeal community composition in hypersaline environments (Maturrano et al., 2006; Youssef et al., 2012; Weigold et al., 2016). *Halobacteria* perform the salt-in strategy for osmoregulation, which generally requires less metabolic energy compared with the synthesis of compatible solutes.

*Trueperaceae* affiliated to the phylum *Deinococcus-Thermus* can be found in the near-surface layers (2–16.5 cm). *Deinococcus*-related bacteria are isolated from hot, arid environments, such as the Tunisian Sahara (Chanal et al., 2006; Theodorakopoulos et al., 2013), hot springs (Albuquerque et al., 2005), as well as radioactive sites (Asker et al., 2011) and industry water (Kämpfer et al., 2008). They are extremely ionizing-radiation-resistant, slightly thermophilic, chemoorganotrophic, and aerobic (Albuquerque et al., 2005; Slade and Radman, 2011).

At depths of 13.5–35.5 cm, between 6 and 17% of the taxa belong to the phylum *Actinobacteria*. Chemo-organotrophic *Actinobacteria* have developed diverse strategies for survival, including sporulation; wide metabolic, degradation capacity; synthesis of secondary metabolites; and various UV repair mechanisms (Ensign, 1978; McCarthy and Williams, 1992). *Actinobacteria* have been isolated from saline soils in Mexico (Valenzuela-Encinas et al., 2009) and described as a dominant phylum in arid environments, such as the Namib Desert (Makhalanyane et al., 2013). In Witpan, actinobacterial sequences can be mainly assigned to the class *Nitriliruptoria* (Ludwig et al., 2012) and the order *Actinomycetes*. In the upper layers, the sequences are related to *Nitriliruptoria*, which has been isolated from soda lake sediments of the Kulunda Steppe (Altai, Russia) (Sorokin et al., 2009). Sorokin et al. (2009) have demonstrated the capability of the species *Niriliruptor alkaliphilus* to degrade complex, naturally occurring nitriles that can be produced during the anaerobic degradation of amino acids (Harper and Gibbs, 1979). Different families of *Actinomycetes* occur infrequently in the depth profile and perform specific mechanisms of adapting to saline and alkaline habitats (Singh et al., 2012). At a depth of 4.5–40 cm, up to 10% of the microbial community can be assigned to the phylum *Planctomycetes*, which comprises aerobic organoheterotrophic *Phycisphaerae* and *Planctomycetaceae*. *Phycisphaerae* were originally isolated from marine algae (Fukunaga et al., 2009) and play an important role in the rhizome-associated concretions (Fernandez et al., 2016). *Planctomycetaceae* are of particular interest for their eukaryotic-like cell structures and their properties of resistance to extreme environmental conditions (Guo et al., 2012).

The deepest layers of Witpan (103–175.5 cm) displayed higher concentrations of acetate and formate as well as higher water content (**Figures 2E–G**). Compared with the overlying sediment layers, the changes in the microbial community structure likely reflect anoxic conditions. *Acetothermia*, *Chloroflexi*, and the family *Bacteroidales* of the phylum *Bacteroidetes* become more abundant. Members of *Bacteroidetes* are adapted to saline conditions and can be observed in a variety of hypersaline systems, including the Atacama Desert (Fernandez et al., 2016; Schulze-Makuch et al., 2018), salterns (Oren et al., 2009), microbial mats (Sørensen et al., 2005), and evaporates (Farías et al., 2014). *Chloroflexi* is also found in hypersaline environments, such as high-saline soils and hypersaline wastewater. Most of the *Chloroflexi* sequences can be found in the deep layers of Witpan and assigned to the class *Dehalococcoidia*. These organohalide-respiring bacteria were first isolated from chloroethene-contaminated terrestrial aquifer environments (Maymo-Gatell, 1997). *Dehalococcoidia* are strictly anaerobic, slow-growing, and highly niche-adapted toward reductive dehalogenation. They might be linked to the accumulation of halogenated organic compounds as a consequence of the humification of plant matter (Hug et al., 2013). Increased concentrations of acetate in the deep layers of Witpan (**Figure 2F**) probably originate from highly active *Acetothermia*, which are chemolithotrophic bacteria that perform acetogenesis as a primary energy- and carbon metabolic pathway (Takami et al., 2012). *Acetothermia* has hardly been described in environmental studies; however, in single-cell amplified genome studies, its potential to adapt to osmotic stress in hypersaline environments has been demonstrated (Nigro et al., 2016). Acetogens utilize the reductive acetyl-CoA pathway (Wood–Ljungdahl pathway) for carbon fixation that is known to be prevalent in the subsurface ecosystems, such as in goldmine boreholes in the Witwatersrand basin in South Africa (Magnabosco et al., 2016). Acetogens only produce acetate as a fermentation product (Ragsdale and Pierce, 2008), but they have a higher threshold for hydrogen than do most methanogens. The archaeal community at deeper levels was mainly formed by euryarchaeal candidate division MSBL1 (previously assigned to *Methanobacteriales*) instead of *Halobacteria*, indicating a community shift due to the changes in the geochemical sediment properties.

DNA-based approaches such as high-throughput sequencing are appropriate for characterizing the microbial communities in detail, while the PLFA method has the advantage of providing quantitative information on the total microbial biomass (Frostegård et al., 2011). The intact phospholipid esters are essential membrane components of living bacterial cells. Since phospholipid esters rapidly degrade after cell death. They are good indicators for viable microorganisms (Zelles, 1999). In this study all PLFAs are summed up as an index of microbial biomass (Quideau et al., 2016; **Figure 4A**). The biomass input decreases with increasing sediment depth that is consistent with other PLFA analyses from soil depth profiles (Bischoff et al., 2013; Stapel et al., 2016). The PLFA profile showed a few peaks at the surface at 45 and 113 cm depth. These peaks are characterized by low species diversity as represented by the Shannon index H. The h-index for Witpan reveals no clear trend, but its diversity index is quite low, similar to the saline shallow lakes of the Monegros Desert in Spain (Casamayor et al., 2013) and the saline sediments such as the Great Salt Plains in Oklahoma (h-Index ∼5) (Youssef et al., 2012).

We were able to show that the carbon-limited and nutrient-poor sediments of Witpan consist mainly of halophilic archaea (e.g., *Halobacteria*) and bacteria (e.g., *Actinobacteria*) as well as microorganisms that are known to be well-adapted to semi-arid conditions (e.g., *Gemmatimonadetes*, *Firmicutes*). Integrating the results from molecular biology with data on soil chemistry suggests a significant correlation between the sulfate- and chloride concentrations in the near-surface layers of Witpan (**Figure 6**). In the deep layers of Witpan, the microbial community structure changes due to increased concentrations of acetate and formate as well as the TOC supply. *Acetothermales* become highly abundant, and acetogenesis might also play an important role.

### Comparative Analysis of Microbial Communities in Kalahari Pans

Microorganisms are involved in different physicochemical and biological processes, such as nutrient cycling, mineralization, and soil aggregation. The microbial diversity is therefore an important component to functioning ecosystems (Keshri et al., 2013). Shared taxa of different study sites can reveal the drivers of the microbial community structure across habitats and help researchers to identify the microbial taxa that have central functions in an ecosystem (Shade and Handelsman, 2012).

We compared the results of high-throughput sequencing data from Witpan in the southern Kalahari with the existing sequencing data on the Omongwa pan, a saline pan in the western Kalahari (Genderjahn et al., 2018). Twenty-nine taxonomic orders are shared between two different pan sediments, and nine phyla can be identified, comprising *Actinobacteria*, *Bacteroidetes*, *Euryarchaeota* (mainly *Halobacteria*), *Firmicutes*, *Gemmatimonadetes*, *Acetothermia*, *Planctomycetes*, and *Proteobacteria* (**Figure 5**). The sequences related to *Actinomycetes* and spore-forming *Bacillus* are highly abundant in both pan sediments. These microorganisms withstand harsh environmental conditions in a state of dormancy or sporulation or as an inactive but viable cell. Once the environmental conditions become more favorable and water has been available for an appropriate amount of time, the cells are able to divide again (Jones and Lennon, 2010; Crits-Christoph et al., 2013).

In contrast to the microbial community of Witpan described above, the Omongwa pan displays two distinct microbial communities in its depth profile. The near-surface community (down to 33 cm) is characterized by a specialized consortium of microorganisms that are attributed to fast-changing conditions and may represent an important refuge for *Gemmatimonadetes* and *Bacteroidetes* as well as for halophilic archaeal *Halobacteria* (**Figure 3B**). In the deeper layers of the Omongwa pan (33 to 105 cm), the microbial diversity decreases compared with the near-surface layers, and only a few species can be identified. The deeper sections are dominated by *Bacillus* (**Figure 3B**), whereas in Witpan, the relative abundance of *Bacillus* is very high in irregular intervals. Representatives of *Firmicutes* are widely distributed in saline habitats, such as a hypersaline crater lake (Paul et al., 2015) or a saline desert in India (Pandit et al., 2014). During sampling, the Omongwa pan was characterized by a distinct halite crust that had been formed due to the evaporation of water and precipitation of chloride and sulfate ions. This salt crust influences the microbial structure and might protect microorganisms against UV radiation. High-throughput sequencing results reveal that the halophilic archaea represent a large proportion of the microbial community, especially in the near-surface sediments of the Kalahari pans (**Figure 3B**). A large percentage of archaea could be observed in the upper layers of the Omongwa pan from 4.5–10.5 cm and in Witpan between 10.5 and 30.5 cm (**Figure 3**). This observation was also described by Maturrano et al. (2006) for salterns of the Peruvian Andes, where archaea dominate over bacteria and a large population of different *Halobacteria* are harbored. The aerobic halophilic archaea form the main microbial biomass in bodies of water with a concentration of sodium chloride approaching saturation, such as soda lakes and crystallizer ponds of solar salterns (Oren, 2002). Different physiological and molecular adaption mechanisms enable the microorganisms to survive under water scarcity. Genderjahn et al. (2017) have described the presence of dialkyl glycerol diethers (DGDs) in the Witpan sediments. DGD lipid membranes might be involved in the ‘salt-in’ strategy and in balancing the osmotic stress by reducing the membrane permeability to H^+^, Na^+^, and other solutes (Dawson et al., 2012). *Halobacteria* can therefore adapt to different salinity conditions and quickly repopulate sediments when the water levels rise and the salinity decreases, such as after rainfall (Kulp et al., 2007).

The relative abundance of *Bacteroidetes*, *Chloroflexi*, and *Proteobacteria* is comparably low in Witpan. The sequences related to *Bacteroidetes Incertae Sedis –* known as *Rhodothermales* and *Cytophagia* – can be classified within *Bacteroidetes* and have recently been identified in alkaline and saline soils (de Leon-Lorenzana et al., 2017) as well as in microbial mats (Fernandez et al., 2016). In pan sediments, the members of thermophilic *Chloroflexi* have been assigned to *Dehalococcoidetes*, which uses organohalide respiration for energy conservation (Kaster et al., 2014). In contrast to the Omongwa pan, *Dehalococcoidetes* mainly occur in the deep anoxic sediments of Witpan. Within the phylum *Proteobacteria*, taxa related to *Ectothiorhodospiraceae*, *Rhodobacteraceae*, and *Rhodospirillales* are particularly prone to occur. Isolates of *Ectothiorhodospiraceae* are found in marine environments as well as in hypersaline and alkaline lakes (Imhoff and Süling, 1996). They are adapted to saline and alkaline growth conditions and grow via anoxygenic photosynthesis by using reduced sulfur compounds, hydrogen, organic compounds, or arsenite as electron donors (Hoeft McCann et al., 2016). Casamayor et al. (2013) have reported the presence of *Rhodobacteraceae* and *Rhodospirillaceae* in shallow saline lakes in the Spanish Monegros Desert. Sequences related to *Planctomycetes* and *Actinobacteria* in Kalahari pan sediments are rare and do not occur frequently.

In contrast, the shared taxa of *Gemmatimonadetes*, *Bacillus*, and *Halobacteria* occur both in Witpan and the Omongwa pan. *Gemmatimonadetes* are always identified with the extreme halophilic *Halobacteria* and *Bacteroidetes*. *Gemmatimonadetes* have not yet been identified as a halophile or halotolerant organism. Studies by DeBruyn et al. (2011) and Fawaz (2013) revealed that a higher abundance of *Gemmatimonadetes* is significantly correlated with low soil moisture. In the investigated pans, *Gemmatimonadetes* were frequently abundant confirming that they are important colonists in arid environments. Additionally, in this study, a high proportion of archaeal sequences could be found, leading to a unique structural depiction of the microbial population in Kalahari pans. *Halobacteria* can also be observed in salt pans on the Namibian coastline, where fog and rainfall are the main sources of water (Cloete, 2015). In general, the metagenomic studies in arid areas reveal a higher prevalence of genes related to osmoregulation, dormancy, and stress response than in non-arid environments. This finding might be a consequence of evolutionary adjustment due to moisture- and hot-stress events (Fierer et al., 2012). In previous studies, Prestel et al. (2008) and Ronca et al. (2015) have described the microbial community studies of the Namib Desert. Sequences of 16S rRNA gene were extracted from Namib Desert surface sands and indicated a high proportion of *Firmicutes*, especially of the genus *Bacillus* (Prestel et al., 2008), which could also be observed in the investigated pan sediments. Furthermore, Prestel et al. (2008) detected members of *Bacteroidetes*, *Planctomycetes*, *Chloroflexi*, and *Deltaproteobacteria*. Makhalanyane et al. (2015) and Ronca et al. (2015) showed a predominance of *Proteobacteria*, *Actinobacteria*, and *Bacteroidetes* within the microbial community in desert systems. These bacterial sequences were found in both pan sediments, but their relative frequency was rather low. One reason for this finding could be technical in nature as high-throughput sequencing allows for a more detailed description of the bacterial and archaeal community.

Our observations point to different localized microbial populations. In the deep layers of Witpan (95–180 cm), the microbial consortia vary widely compared with the upper layers. Anaerobic archaea and bacteria in the deep layers of Witpan dominate the microbial community structure which may be due to lack of oxygen. Additionally, the archaeal community shifts from *Halobacteria* to MSBL1. The relative abundance of sequences related to *Acetothermales* ranges from 10–44%. Increased concentrations of acetate (**Figure 2F**) suggested highly active *Acetothermales*.

High-throughput sequencing allows for deep insights to be made into the microbial community structure of Kalahari pans. When comparing two different study sites, saline- and desiccation-tolerant microorganisms were emphasized and described as dominating taxa. Their present existence in pan sediments is closely related to the surface processes that control water, substrate-, and nutrient availability.

## Conclusion

Despite the possibility of the occurrence of novel microorganisms in hypersaline environments with high economic and industrial potential, only a few detailed reports on the microbial diversity in the Kalahari have been produced thus far. Our study provides new insights into the archaeal and bacterial diversity of desert ecosystems. In both the continental pans, halophilic and desiccation-tolerant taxa were found. Analyses of high-throughput sequencing data from two different pan sediments identified a core community dominated by *Firmicutes*, *Gemmatimonadetes*, and *Halobacteria*. The high abundance of halophilic archaea and their influence on biogeochemical cycles remain largely unexplored yet represent a major aspect of the desert ecosystem. The deep, anoxic layers of Witpan are characterized by a special consortium of microorganisms. Higher concentrations of acetate indicate the presence of highly active *Acetothermia*, which perform acetogenesis. In addition, *Dehalococcoides* are also present in the deeper layers of Witpan. This new knowledge might be useful for the treatment of wastewater. An investigation of novel niches that harbor dehalorespiring microorganisms could broaden our knowledge of organohalide biodegradation applications.

## Author Contributions

SG performed all experiments, interpreted the results, and wrote the manuscript. MA substantially contributed to data interpretation and revised the manuscript. KM and SG sampled during the field campaign. KM contributed to the development of the concept and helped with valuable discussion. FH processed next generation sequencing data. DW provided financial and technical support and revised the manuscript.

## Conflict of Interest Statement

The authors declare that the research was conducted in the absence of any commercial or financial relationships that could be construed as a potential conflict of interest.
