# Supplementary material for: Desiccation- and Saline-Tolerant Bacteria and Archaea in Kalahari Pan Sediments
Source: Front Microbiol. 2018 Sep 20;9:2082. doi: 10.3389/fmicb.2018.02082 (PMC6158459; doi:10.3389/fmicb.2018.02082)
Supplement: Supplementary file 1 [file Table_1.DOCX]

Supplementary Material

**Desiccation- and saline-tolerant bacteria and archaea in**

**Kalahari pan sediments**

Steffi Genderjahn*, Mashal Alawi, Kai Mangelsdorf, Fabian Horn, Dirk Wagner

Correspondence: Steffi Genderjahn: [steffi.genderjahn@gfz-potsdam.de](mailto:steffi.genderjahn@gfz-potsdam.de)

**Table S1:** Analytical settings for the determination of ion concentration in leached water.

|  | **Inorganic anions** | **Organic acids** |
| --- | --- | --- |
| **Instrument** | Sykam IC | Dionex ICS 3000 |
| **Column** | LCA A14 | As11Hc 2x250 mm |
| **Oven temperature** | 65 °C | 35 °C |
| **Suppressor** | SAMSTM, SeQuant, Sweden | ASRS Ultra II 2 mm |
| **Detector** | SYKAM S3115 conductivity detector | Dionex conductivity detector |
| **Mobile phase** | 12.5 ml/l sodium carbonate (Na2CO3) (0.5 M) + 1 ml/l Modifier (1 g 4-hydroxybenzonitrile in 50 ml methanol) | KOH in varying concentrations |
| **Elution** | isocratic | gradient |
| **Eluent flow** | 1 ml/min | 0,38 ml/min |
