# Supplementary material for: Desiccation- and Saline-Tolerant Bacteria and Archaea in Kalahari Pan Sediments
Source: Front Microbiol. 2018 Sep 20;9:2082. doi: 10.3389/fmicb.2018.02082 (PMC6158459; doi:10.3389/fmicb.2018.02082)
Supplement: Supplementary file 2 [file Table_2.DOCX]

Supplementary Material

**Desiccation- and saline-tolerant bacteria and archaea in**

**Kalahari pan sediments**

Steffi Genderjahn*, Mashal Alawi, Kai Mangelsdorf, Fabian Horn, Dirk Wagner

Correspondence: Steffi Genderjahn: [steffi.genderjahn@gfz-potsdam.de](mailto:steffi.genderjahn@gfz-potsdam.de)

**Table S2:** Sequencing read counts preprocessing.

|  |  |  | read counts preprocessing | | | |
| --- | --- | --- | --- | --- | --- | --- |
|  | Sample name | depth [cm] | After sorting | After merging | After trimming | After chimera removal |
| Omongwa pan | G014572 | 0.25 | 33040 | 32634 | 32450 | 30761 |
|  | G014572_2 | 0.25 | 76520 | 75918 | 75558 | 72596 |
|  | G014573 | 1.75 | 68001 | 67589 | 67043 | 64624 |
|  | G014573_2 | 1.75 | 123241 | 122608 | 121739 | 117385 |
|  | G014574 | 4.5 | 61505 | 61160 | 60686 | 59058 |
|  | G014574_2 | 4.5 | 96078 | 95559 | 94858 | 92262 |
|  | G014575 | 7.5 | 48132 | 47858 | 47466 | 43278 |
|  | G014575_2 | 7.5 | 91297 | 90858 | 90282 | 86522 |
|  | G014576 | 10.5 | 52232 | 51972 | 51566 | 49947 |
|  | G014576_2 | 10.5 | 84411 | 83983 | 83383 | 81150 |
|  | G014577 | 13.5 | 49862 | 49604 | 49225 | 45639 |
|  | G014577_2 | 13.5 | 80560 | 80177 | 79655 | 77569 |
|  | G014579 | 23 | 63751 | 63500 | 63191 | 55793 |
|  | G014579_2 | 23 | 71673 | 71426 | 71074 | 60471 |
|  | G014580 | 28 | 35087 | 34976 | 34842 | 28453 |
|  | G014581 | 32.5 | 50419 | 50291 | 50152 | 48714 |
|  | G014581_2 | 32.5 | 13770 | 13720 | 13681 | 12397 |
|  | G014582 | 37.5 | 25931 | 25842 | 25767 | 25253 |
|  | G014582_2 | 37.5 | 33109 | 33032 | 32926 | 32297 |
|  | G014583_2 | 42.5 | 22847 | 22775 | 22674 | 22169 |
|  | G014584 | 45.5 | 66390 | 66167 | 65887 | 64298 |
|  | G014584_2 | 45.5 | 90692 | 90382 | 89997 | 87472 |
|  | G014587_2 | 65 | 54441 | 53946 | 53714 | 50767 |
|  | G014588 | 75 | 27153 | 27056 | 26957 | 26450 |
|  | G014588_2 | 75 | 54526 | 54351 | 54130 | 52313 |
|  | G014590_2 | 95 | 59409 | 59084 | 58859 | 56865 |
|  | G014591 | 95 | 33160 | 33001 | 32889 | 32098 |
|  | G014591_2 | 105 | 64465 | 64131 | 63891 | 61293 |
| Witpan | G014614 | 1 | 133132 | 132557 | 131797 | 128407 |
|  | G014614_2 | 1 | 85291 | 84956 | 84440 | 81030 |
|  | G014615 | 2 | 107228 | 106727 | 106015 | 102891 |
|  | G014615_2 | 2 | 95018 | 94574 | 93874 | 91000 |
|  | G014616 | 4.5 | 86917 | 86522 | 85815 | 81423 |
|  | G014616_2 | 4.5 | 93203 | 92738 | 91985 | 88996 |
|  | G014617 | 7 | 51940 | 51689 | 51375 | 48282 |
|  | G014617_2 | 7 | 65325 | 65052 | 64627 | 61975 |
|  | G014618 | 8.5 | 120893 | 120304 | 119103 | 116933 |
|  | G014618_2 | 8.5 | 85856 | 85438 | 84859 | 82202 |
|  | G014619 | 10.5 | 132267 | 131736 | 130733 | 128797 |
|  | G014619_2 | 10.5 | 61614 | 61246 | 60612 | 59388 |
|  | G014620 | 13.5 | 109355 | 108839 | 107952 | 105755 |
|  | G014620_2 | 13.5 | 87390 | 87023 | 86507 | 80338 |
|  | G014621 | 16.5 | 105262 | 104776 | 103918 | 99090 |
|  | G014621_2 | 16.5 | 96628 | 96237 | 95648 | 91863 |
|  | G014622 | 19.5 | 110303 | 109626 | 108876 | 105715 |
|  | G014622_2 | 19.5 | 55117 | 54894 | 54620 | 52794 |
|  | G014623 | 22.5 | 60773 | 60498 | 59942 | 59003 |
|  | G014623_2 | 22.5 | 130488 | 129968 | 129101 | 126231 |
|  | G014624 | 26 | 72251 | 71943 | 71359 | 67727 |
|  | G014624_2 | 26 | 69722 | 69453 | 68992 | 67064 |
|  | G014625 | 30.5 | 32637 | 32272 | 32044 | 31277 |
|  | G014625_2 | 30.5 | 51953 | 51751 | 51419 | 48727 |
|  | G014626 | 35.5 | 120498 | 119871 | 118724 | 113906 |
|  | G014626_2 | 35.5 | 59043 | 58809 | 58434 | 56714 |
|  | G014627 | 40.5 | 108842 | 108056 | 107249 | 102182 |
|  | G014627_2 | 40.5 | 45362 | 45121 | 44798 | 39120 |
|  | G014628 | 45.5 | 22007 | 21846 | 21683 | 19496 |
|  | G014628_2 | 45.5 | 90004 | 89497 | 88925 | 85728 |
|  | G014629 | 50.5 | 94257 | 93776 | 92940 | 88749 |
|  | G014629_2 | 50.5 | 122710 | 122128 | 121137 | 118114 |
|  | G014630 | 55.5 | 116384 | 115891 | 115066 | 109660 |
|  | G014630_2 | 55.5 | 129554 | 129054 | 128032 | 122974 |
|  | G014631 | 60.5 | 75894 | 75530 | 75003 | 72999 |
|  | G014631_2 | 60.5 | 80350 | 79975 | 79564 | 76289 |
|  | G014632 | 65.5 | 65999 | 65433 | 65012 | 62701 |
|  | G014632_2 | 65.5 | 70408 | 70081 | 69628 | 67709 |
|  | G014633 | 73 | 94458 | 93983 | 93157 | 89277 |
|  | G014633_2 | 73 | 120667 | 120136 | 119197 | 116866 |
|  | G014634 | 83 | 100398 | 99957 | 99183 | 95824 |
|  | G014634_2 | 83 | 118545 | 117951 | 116820 | 113974 |
|  | G014635 | 92 | 62353 | 62088 | 61790 | 59700 |
|  | G014635_2 | 92 | 57464 | 57268 | 56934 | 55273 |
|  | G014636 | 95 | 37661 | 37522 | 37280 | 35287 |
|  | G014636_2 | 95 | 106818 | 106454 | 105793 | 102040 |
|  | G014637 | 103 | 51700 | 51392 | 51171 | 23710 |
|  | G014638 | 113 | 32327 | 32154 | 31970 | 31220 |
|  | G014638_2 | 113 | 49024 | 48871 | 48658 | 47110 |
|  | G014639 | 123 | 91694 | 91275 | 90684 | 65408 |
|  | G014639_2 | 123 | 36714 | 36582 | 36359 | 34763 |
|  | G014640 | 133 | 106181 | 105541 | 104705 | 92682 |
|  | G014640_2 | 133 | 129026 | 128527 | 127594 | 123551 |
|  | G014641_2 | 145.5 | 109324 | 108808 | 107993 | 102145 |
|  | G014642 | 160.5 | 61534 | 61326 | 61023 | 36511 |
|  | G014642_2 | 160.5 | 90273 | 89999 | 89605 | 70142 |
|  | G014643 | 175.5 | 41720 | 41578 | 41390 | 40188 |
|  | G014643_2 | 175.5 | 61705 | 61371 | 61123 | 57545 |
