# Supplementary material for: Desiccation- and Saline-Tolerant Bacteria and Archaea in Kalahari Pan Sediments
Source: Front Microbiol. 2018 Sep 20;9:2082. doi: 10.3389/fmicb.2018.02082 (PMC6158459; doi:10.3389/fmicb.2018.02082)
Supplement: Supplementary file 3 [file Data_Sheet_1.PDF]

Supplementary Material

Desiccation- and saline-tolerant bacteria and archaea in Kalahari pan sediments

Steffi Genderjahn*, Mashal Alawi, Kai Mangelsdorf, Fabian Horn, Dirk Wagner

Correspondence: Steffi Genderjahn: steffi.genderjahn@gfz-potsdam.de

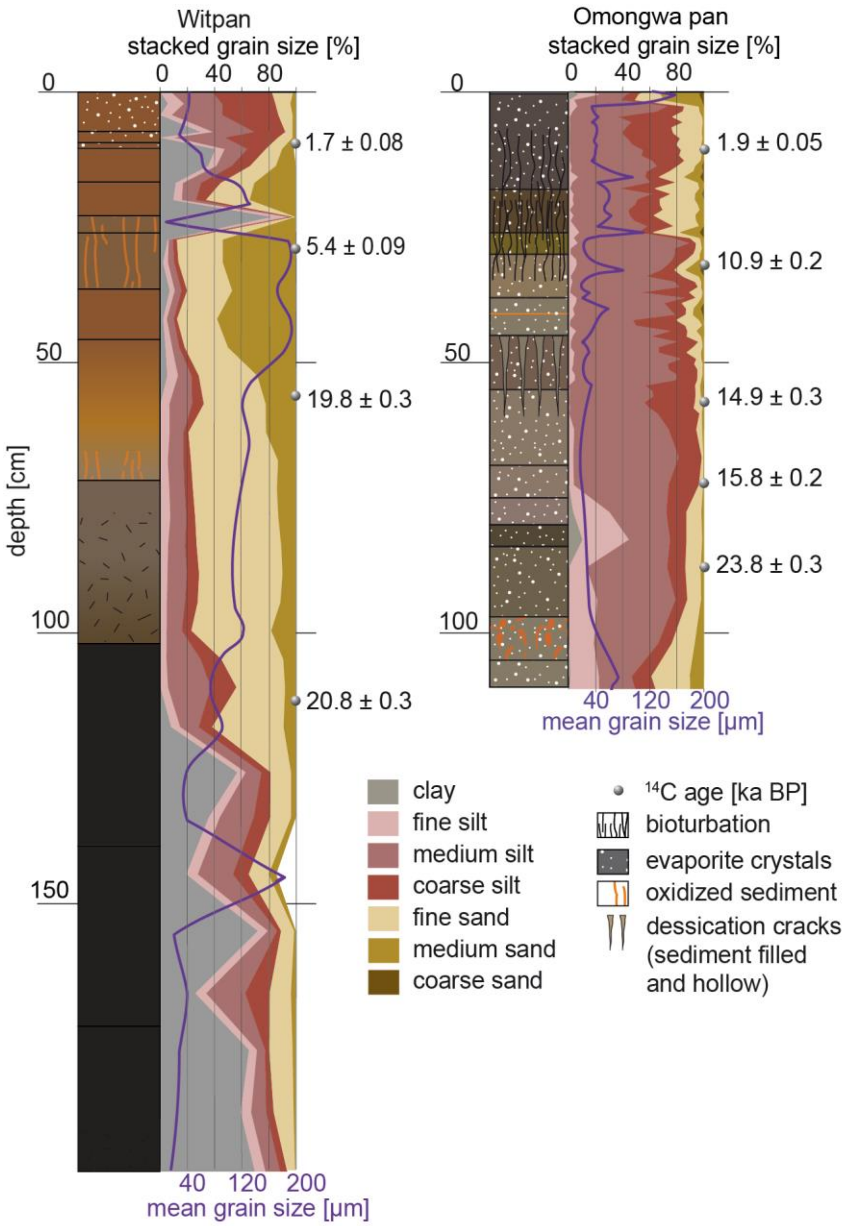

Figure S1: Sedimentological profile with stacked grain size distribution and mean grain size (0 – 250 µm; violet line) of Witpan and Omongwa pan. Sediment colors refer to the Munsell soil color chart. After Schüller et al. (2018)
