# Supplementary material for: Desiccation- and Saline-Tolerant Bacteria and Archaea in Kalahari Pan Sediments
Source: Front Microbiol. 2018 Sep 20;9:2082. doi: 10.3389/fmicb.2018.02082 (PMC6158459; doi:10.3389/fmicb.2018.02082)
Supplement: Supplementary file 4 [file Data_Sheet_2.PDF]

Supplementary Material

Desiccation- and saline-tolerant bacteria and archaea in Kalahari pan sediments

Steffi Genderjahn*, Mashal Alawi, Kai Mangelsdorf, Fabian Horn, Dirk Wagner

Correspondence: Steffi Genderjahn: steffi.genderjahn@gfz-potsdam.de

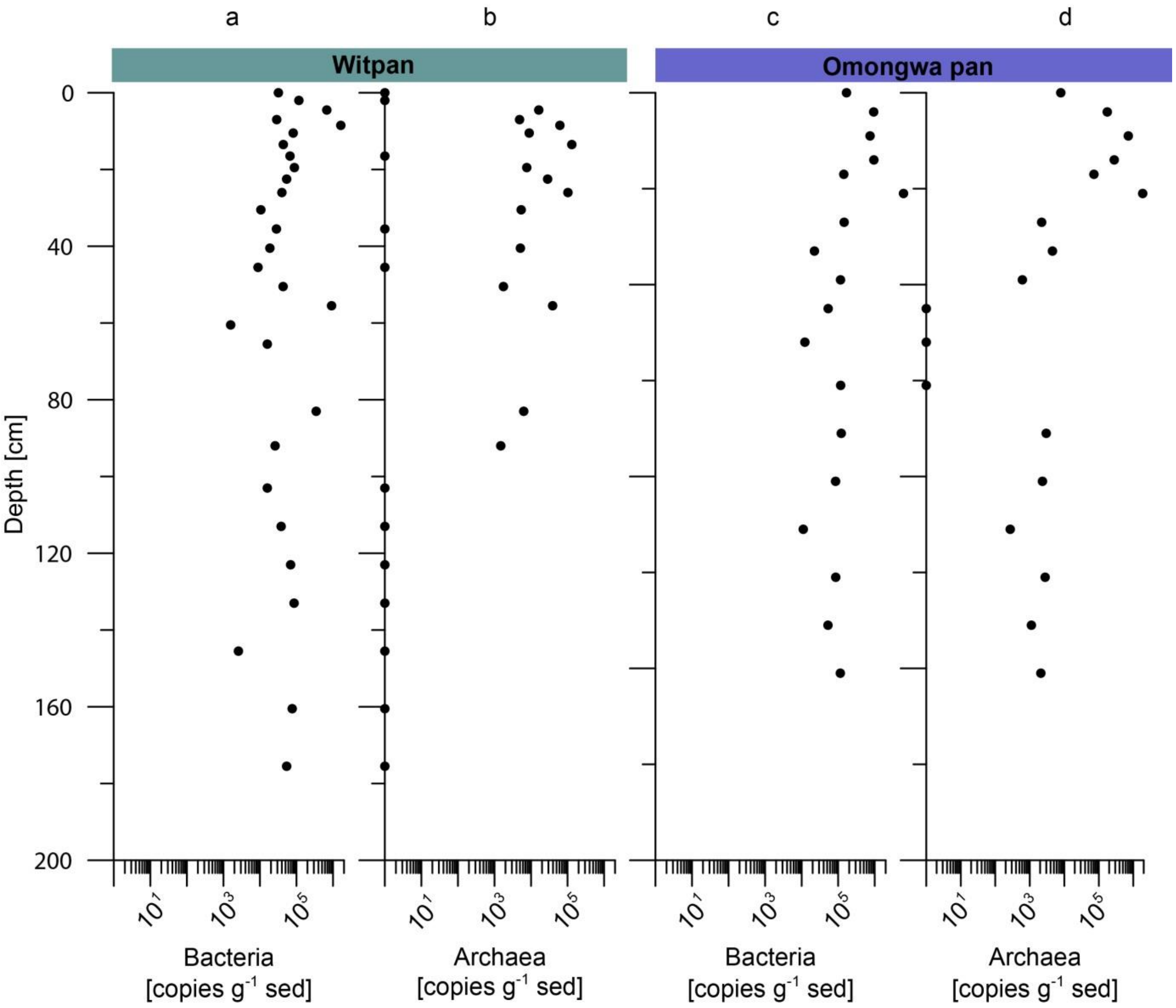

Figure S2: qPCR results of Witpan (a + b) and Omongwa pan (c + d). (a + c) abundance of bacterial small subunit ribonucleic acid genes (copies per gram sediment), (b + d) abundance of archaeal small subunit ribonucleic acid genes (copies per gram sediment, shown on a log scale)
